# Coronary Chronic Total Occlusion Revascularization: When, Who and How?

**DOI:** 10.3390/jcm13071943

**Published:** 2024-03-27

**Authors:** Elisabetta Ricottini, Federica Coletti, Annunziata Nusca, Nino Cocco, Andrea Corlianò, Alessandro Appetecchia, Rosetta Melfi, Fabio Mangiacapra, Paolo Gallo, Raffaele Rinaldi, Francesco Grigioni, Gian Paolo Ussia

**Affiliations:** 1 Cardiology Unit, Fondazione Policlinico Universitario Campus Bio-Medico, 00128 Rome, Italy; e.ricottini@policlinicocampus.it (E.R.); federica.coletti@unicampus.it (F.C.); n.cocco@policlinicocampus.it (N.C.); andrea.corliano@unicampus.it (A.C.); a.appetecchia@unicampus.it (A.A.); r.melfi@policlinicocampus.it (R.M.); f.mangiacapra@policlinicocampus.it (F.M.); p.gallo@policlinicocampus.it (P.G.); r.rinaldi@policlinicocampus.it (R.R.); f.grigioni@policlinicocampus.it (F.G.); g.ussia@policlinicocampus.it (G.P.U.); 2 Cardiology Unit, Department of Medicine, Campus Bio-Medico University of Rome, 00128 Rome, Italy

**Keywords:** coronary chronic total occlusions, percutaneous coronary interventions, complex coronary artery disease

## Abstract

Coronary chronic total occlusions (CTO) are an increasingly frequent entity in clinical practice and represent a challenging percutaneous coronary intervention (PCI) scenario. Despite data from randomized trials that have not yet demonstrated a clear benefit of CTO recanalization, the widespread of CTO-PCI has substantially increased. The improvement in operators’ techniques, equipment, and training programs has led to an improvement in the success rate and safety of these procedures, which will represent an important field of future development of PCI. The present review will summarize clinical outcomes and technical and safety issues of CTO revascularization with the aim to guide clinical daily cath-lab practice.

## 1. Introduction

A chronic total occlusion (CTO) is a complete luminal occlusion of a coronary artery with a duration of ≥3 months. The prevalence of CTO among patients with coronary artery disease (CAD) undergoing coronary angiography is 16–18% in large clinical registries [1,2,3]. Despite the high prevalence of chronic occlusions, CTO revascularization has historically represented a limited percentage of the total cases of percutaneous coronary interventions (PCI). Large registries showed that most of the patients receiving an angiographic diagnosis of CTO were treated with optimal medical therapy (OMT), while only a minority of these subjects underwent PCIs or coronary artery bypass graft surgery (CABG) [4,5]. The traditional preference for OMT in CTO patients was mainly related to low procedural success rates, high incidence of complications, high doses of contrast medium, and high radiation dose. Another relevant aspect is the perceived lack of clinical benefit [5]. However, in recent years, the advances in CTO recanalization techniques and equipment, together with the higher operators’ expertise, have led to a growing scientific and clinical interest with increased diffusion of CTO PCI. The aim of this review is to summarize data and recent advances in CTO revascularization to provide an update to support decision-making in everyday clinical practice.

## 2. Methodology

We conducted a literature search through PubMed, Embase, EBSCO, Cochrane database of systematic reviews, and Web of Science from its inception up to 18 September 2023 using the following search keywords in various combinations: ‘chronic total occlusion’, ‘percutaneous coronary intervention’, ‘recanalization’, ‘revascularization’ and combinations between them. We also reviewed references of prior systematic reviews, meta-analyses, and abstracts from major cardiology congresses. Editorials, case series, and ongoing trials without results were excluded. We considered only articles in the English language. References of the selected studies were also thoroughly screened with a “snowball” approach. Two investigators (E.R., F.C.) independently reviewed the studies to determine their eligibility. The entire list of references was reviewed, and disagreements were resolved via consensus.

## 3. CTO Revascularization: When? Who?

### 3.1. Current Evidence and Future Directions

**Figure 1:**
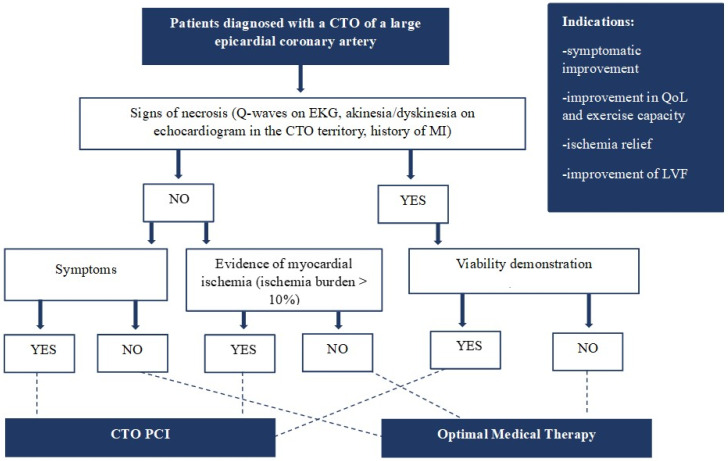
**Diagnostic and therapeutic work-up in patients presenting with a chronic total occlusion.** CTO: chronic total occlusion; LVF: left ventricular function; MI: myocardial infarction; QoL: quality of life; PCI: percutaneous coronary intervention.

Randomized controlled trials (RCTs) comparing CTO recanalization to medical therapy failed to show mortality reduction in CTO patients (Table 1). However, most of these trials were limited by some bias, such as the small number of patients, the significant crossover between the two arms, the treatment of non-CTO lesions in both groups and the relatively short period of follow-up. The only trial that demonstrated a decrease in major adverse cardiac events (MACEs) was the REVASC trial [6]. However, only three deaths were registered in the one-year follow-up, and most of the MACEs were represented by target CTO vessel revascularization incidents. Although the disappointing results on MACEs, some trials (EURO CTO) and several controlled registries showed improvement in quality of life and symptoms, reduction of the ischemic burden, and upgrading in left ventricular function (LVF) in patients undergoing CTO recanalization [7,8,9,10,11,12,13]. For instance, a meta-analysis by Joyal et al. demonstrated that the incidence of residual or recurrent angina was significantly reduced in patients with successful CTO recanalization compared to those with failed attempts [14]. The OPEN CTO registry showed that dyspnoea, present in up to 50% of CTO patients, improved in 70% of cases and disappeared in over 42% after successful recanalization [15]. Moreover, retrospective data indicated a potential benefit of successful CTO recanalization in electric stabilization and decreased arrhythmic burden [16,17]. Based on this evidence, we can assume that in the current era, the attempt of a CTO revascularization will be appropriate when there is a high possibility of achieving reliable results (symptom relief, reduction of the ischemic burden, and improvement in LVF) with minimizing the risk of contrast media and radiation exposure (Figure 1). Moreover, a successful CTO PCI program should include high operators’ experience and adequate availability of materials and supporting tools.

Whether CTO revascularization improves hard clinical outcomes remains to be proven. Future trials will try to supply definitive responses in this section. Specifically, the ISCHEMIA-CTO (NCT03563417) trial will include more than 1500 patients randomized to CTO PCI versus OMT. The study hypothesis is that in asymptomatic patients with >10% myocardial ischemia, CTO PCI is superior to OMT in reducing MACEs. Similarly, the NOBLE-CTO will examine all-cause mortality and quality of life in patients undergoing either PCI CTO or OMT as the initial strategy (NCT03392415). ORBITA-CTO will compare PCI CTO vs. placebo procedure in reducing angina in patients with a previous 3-months OMT (NCT05142215). CTO-HF trial (NCT05632653) will investigate if CTO revascularization improves survival and heart failure (HF) rehospitalizations compared to OMT.

### 3.2. Patients with Symptoms and Myocardial Ischemia

Symptoms are one of the main indications for a CTO recanalization procedure. In this regard, it is relevant to know that CTO patients mostly report atypical symptoms: symptoms are described as typical angina only in a few cases. More frequently, patients complain of breathlessness or fatigue, which are often assigned to non-cardiac conditions. Moreover, given the chronic condition, patients experienced a slow and progressive reduction of physical performance, as also objectified by some studies performing cardiorespiratory tests in CTO patients [18,19]. Another relevant aspect is the evidence of myocardial ischemia in the distribution area of the occluded coronary artery. Several studies showed that, in most cases, collateral circulation is unable to maintain adequate myocardial perfusion [20,21]. Thus, developing collateral circulation may avoid myocardial necrosis but not ischemia. Therefore, well-developed collaterals should not influence the indication for CTO recanalization. Regarding inducible myocardial ischemia, traditionally, a percentage of ischemic myocardium > 10% was associated with a poor prognosis [22]. However, It is important to underline that most of the studies investigating myocardial ischemia are not CTO-focused. Recently, the ISCHEMIA (International Study of Comparative Health Effectiveness with Medical and Invasive Approaches) trial showed that in patients with demonstrated myocardial ischemia (including those with CTO lesion), revascularization with PCI or CABG is not superior to OMT for reducing MACEs. Specific to CTO lesions, it is important to underline that most CTOs in the ISCHEMIA trial were not revascularized. The incomplete revascularisation in the presence of CTOs may have played a role in this disappointing outcome [20,21,22,23]. Moreover, in the ISCHEMIA trial, some patients with CTOs (that were not revascularized) received PCI to non-ischemic non-CTO vessel territories. Therefore, the results of the ISCHEMIA trial cannot be strictly applied to CTO patients [23].

### 3.3. Patients with Reduced Left Ventricular Ejection Fraction (LVEF)

CTO recanalization has a solid rationale in patients with reduced LVEF and wall motion abnormalities of the myocardium subtended by the occluded artery. However, the diagnostic work-up in these patients should include the assessment of myocardial viability. In the absence of signs of myocardial viability, no improvement of LVEF is expected, and CTO revascularization is considered inappropriate (Figure 1). On the other hand, myocardial viability before CTO revascularization predicts the recovery of regional and global LVF [24,25]. In addition, the more the stunned but vital myocardial area, the greater the LVF recovery [26]. Myocardial viability can be assessed using a stress echocardiogram or CMR. Specifically, CMR represents the gold standard for assessing myocardial viability and fibrosis. The extension of LGE on CMR is directly related to the expected positive remodeling of the left ventricle and, consequently, to the improvement of LVEF [24,25,26].

### 3.4. Patients with Acute Coronary Event

The presence of CTO in non-culprit coronary arteries has been reported in about 10% of patients presenting with ST-segment elevation myocardial infarction (STEMI) [1]. Concomitant CTO in STEMI patients increases short-term and long-term mortality, mainly when an infarct-related artery (IRA) provides collateral circulation for a chronic occluded artery [27]. Data in the literature about the impact of a CTO PCI in patients presenting with ACS are rare. In fact, the clinical scenario of ACS is definitely more complex compared to the chronic coronary syndrome. Patients with STEMI often present with hemodynamic instability, which precludes complex coronary interventions in the acute setting. The few existing data are also conflicting. The randomized trial EXPLORE, which examined the potential gain in LVEF due to non-IRA CTO re-canalization within one week after STEMI, showed no significant benefit in patients undergoing CTO PCI compared to patients treated with OMT [28]. Conversely, a subsequent sub-study of the EXPLORE trial demonstrated an improvement in regional LVF after CTO recanalization in STEMI patients [29]. Moreover, the effect of CTO PCI on regional LVF might be associated with other positive effects, such as the prevention of arrhythmias, given the characteristic electrical instability of patients presenting with ACS [29]. We can assume that the beneficial effects of a CTO recanalization in non-IRA may be of particular importance in a high-risk group with an infarct-related donor artery and a collateral origin in the proximal position to the acute occlusion. However, more data are needed to support CTO PCI in patients with acute coronary events and to identify the best timing of intervention.

## 4. CTO Revascularization: How?

### 4.1. Procedural Planning

Accurate procedural planning is mandatory when CTO recanalization is considered appropriate. Firstly, a good diagnostic angiogram is crucial to study the proximal cap, the distal one, the CTO lesion length, and the collateral circulation [30,31]. This analysis is preliminary to the choice of revascularization strategy. Coronary computed tomography angiography (CCTA) can represent an additional resource for procedural planning [32].

Planning vascular access is another important step. Double arterial access is often recommended, mainly when collateral circulation originates from the contralateral coronary artery. Dual coronary angiography facilitates CTO PCI planning and, additionally, during the procedure, it facilitates guiding crossing attempts by helping to determine the guidewire position. In case of complex occlusions in patients with a history of previous CABG, triple access may be needed since two or three different sources of collateral circulation might be present.

The need for mechanical circulatory support should be considered in high-risk patients, such as patients with severe reduction of LVEF and the last remaining vessel.

A careful check of renal function is mandatory, particularly in diabetic patients. The ratio of contrast medium to creatinine clearance should generally be <3 [33].

The evaluation of the anatomical complexity of a CTO is another relevant aspect of procedural planning. In this regard, multiple complexity scores have been developed. These scores can predict procedure duration and succession rates and help select patients whom dedicated CTO operators should manage (Table 2) [34,35,36,37,38,39,40].

### 4.2. Revascularization Strategies

When discussing the revascularization strategy, we primarily refer to the choice of an anterograde, retrograde, or combined approach.

### 4.3. Anterograde Recanalization

We can divide the anterograde wiring (AW) into three main groups: (I) Antegrade wire escalation/de-escalation; (II) Parallel wiring; (III) Antegrade dissection and re-entry.

#### 4.3.1. Antegrade Wire Escalation/De-Escalation

In the case of an unambiguous proximal cap, good quality of the distal and proximal cap and vessel course and short occlusion length (<20 mm) antegrade wiring is the first-choice technique [41,42,43]. In some cases, in the presence of a stent that indicates the arterial path, it can also be used for longer occlusions [44]. This type of CTO can be easily crossed with this technique and has minor complications. In the context of wiring escalation, the use of a microcatheter is required to avoid dissections or subintimal tracking of the coronary vessel proximal to the occlusion. Furthermore, the assistance of a microcatheter is necessary to rapidly interchange specialized guidewires and, consequently, to facilitate progression within the occlusion. Guidewires for the AW can be divided into three categories (Table 3) [40,41,42,43,44].

#### 4.3.2. Parallel Wiring

**Figure 2:**
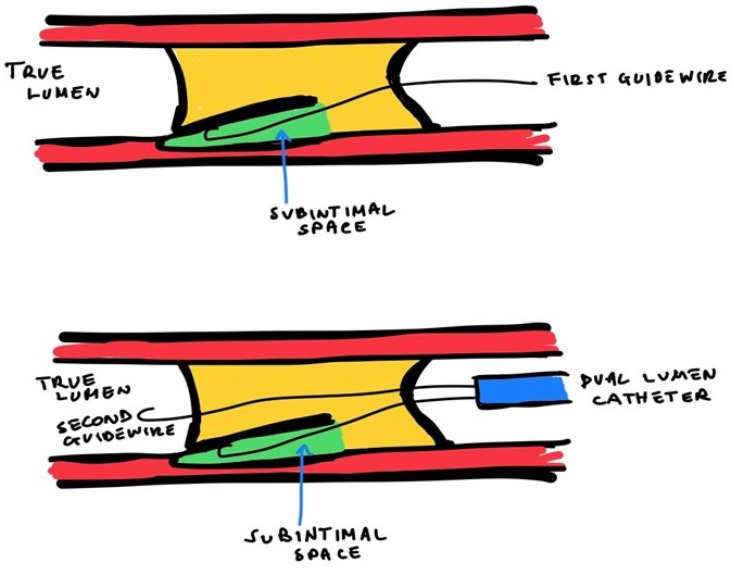
**Parallel wiring technique**.

In case of direct AW failure because of the wire entering the subintimal space, the parallel wiring technique can be used. In this technique, the first wire, which is located in the subintimal space, is used as a reference, while a second guidewire is introduced within the occlusion, parallel to the first one, to penetrate the distal cap (Figure 2).

#### 4.3.3. Antegrade Dissection and Re-Entry (ADR)

In case of tortuosity that makes the vessel course uncertain, or in case of severe calcifications, the operator can advance the wire into the subintimal space [41,42,43]. Using the “knuckle technique” it is possible to navigate in subintimal space with a hydrophilic wire with a J-loop configuration to allow a blunt dissection between the anatomical planes of the vessel using the principle of differential longitudinal and tangential resistance [45] and then re-enter in the true lumen with a dedicated device (i.e., Stingray balloon, dual lumen microcatheters [DLMC]).

The Stingray balloon device (a flat balloon with lateral exit ports that allow an easy and controlled re-entry inside the true lumen from the subintimal dissection obtained by the wire) is brought inside the subintimal space distal to the occluded tract. After the inflation of the balloon enhances the stability of the position, a high tip-load guidewire is used to re-enter into the true lumen [46]. As an alternative, the monorail port of the DLMC is loaded over the wire inside the subintimal space, while to achieve the re-entry into the distal true lumen from the occlusion is used a stiff high tip load guidewire loaded on the over the wire port [47]. It is not recommended to use this technique in the case of left anterior descending coronary artery CTO with multiple branches (septal and diagonal) because of the risk of occlusion.

**Figure 3:**
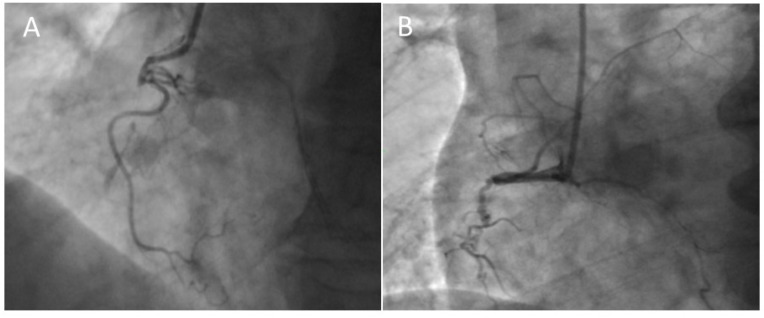
**Proximal CTO cup.** (**A**) Tapered cup with microchannel with a high chance to anterograde success. (**B**) Stump cup with bridging collaterals with low chance to anterograde success.

The proximal cap ambiguity represents a main challenge in the antegrade technique. It means the impossibility of determining the proximal entry point into a CTO because of vessel tortuosity, the presence of side branches (SB), or ostial CTO (Figure 3).

- If there is a SB at the occlusion site, antegrade IVUS-guided wiring can be helpful. The ultrasound probe is conducted into the SB to visualize the ostium of the CTO, and then the operator can perform the cap puncture under IVUS visualization.
- In case of the absence of a SB, the operator can advance the IVUS in a dissection plan near the cap of the CTO. To make the dissection, the operator safely starts the advancing of the probe into the subintimal space within the unknown arterial course (“move-the-cap” techniques) [48]:
  (1) Balloon-assisted subintimal entry (BASE): a balloon is inflated proximally to the CTO cap to have a small disruption of the intimal layer, allowing wire passage in this space [49];
  (2) Carlino technique: the tip of a microcatheter proximally to the CTO cap, the operator can perform a microinjection of contrast determining a focal hydraulic dissection [50];
  (3) “Scratch-and-go” technique: a puncture the extra plaque space proximally to the cap of the CTO with a high tip-load penetrative wire. Therefore, the high tip-lead guidewire is exchanged via a microcatheter for a polymeric guidewire for subintimal advancement [48].

### 4.4. Retrograde Recanalization

If all the anterograde recanalization techniques are not feasible due to proximal cap ambiguity and poor distal vessel quality, the retrograde way should be considered. Such an approach could be the only possibility to resolve when there is a bifurcation in the distal cap or when the anterograde way is not feasible. As for the anterograde wiring, the retrograde way can be performed via retrograde wiring inside the obstruction or via retrograde dissection and re-entry [51,52,53].

#### 4.4.1. Retrograde and Anterograde Lumen Connection

**Figure 4:**
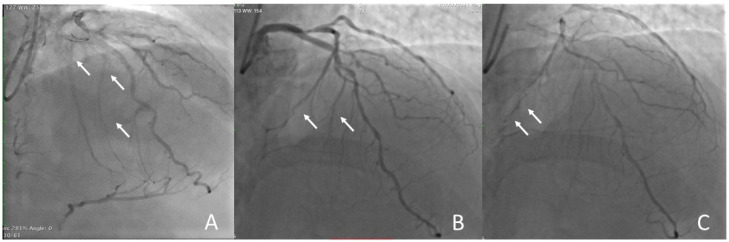
**Choice of collaterals for retrograde approach.** (**A**) Good interventional septal collaterals from LAD to RCA. (**B**) No-interventional septal collaterals: there is no connection with the DP of RCA. (**C**) No-interventional epicardial collaterals: connection with PL ramus of RCA but small and tortuous at high risk of perforations. The arrows show the collateral vessels.

The first and most important step for retrograde recanalization is the selection of the collateral vessel. The operator must choose the best collateral vessel based on tortuosity, size, and his/her experience. If the operator should have both septal and epicardial collaterals at his/her disposal, the epicardial collaterals should be avoided due to the higher risk of causing tamponade by bleeding directly into the pericardial space, preferring the septal collaterals (Figure 4).

After the collateral wiring, the operator must advance a microcatheter into the distal coronary near the distal cap. In the case of epicardial collateral, wiring requires specific care to avoid overstretching and damage, choosing a tapered thin microcatheter (i.e., Caravel—Asahi Intecc or Turnpike L.P.—Teleflex) [42]. The best approach in a retrograde way is crossing the guidewire through the occlusion supported by a microcatheter from the distal true lumen to the proximal lumen. If the guide wire does not proceed towards the proximal cap or it is not possible to identify the right path of the vessel, the operator can choose to use the reverse CART technique. In this technique, two wires, antegrade and retrograde, are advanced within the occlusive lesion, and one wire is moved to meet the other wire. To find a route into the proximal vessel, a balloon is advanced on the antegrade wire and dilated in the occluded segment to find a route into the proximal vessel to make the retrograde wire follow the right direction [54].

#### 4.4.2. Retrograde Dissection Re-Entry (RDR)

This technique is based on the advance of antegrade and retrograde wires until they overlap inside the occlusion. This procedure is required when the vessel course is ambiguous and the occluded segment is long [43].

#### 4.4.3. Externalization of the Guidewire

When the wire has crossed the entire lesion, it will be necessary to externalize the wire using the antegrade guide catheter. A guide catheter extension can help to facilitate the wire externalization in the case of incorrect alignment of the guiding catheter and the proximal vessel due to disease of the segment proximal to the occlusion. Suppose the retrograde microcatheter does not cross the occlusion or is short. In that case, the retrograde wire can be inserted in the antegrade microcatheter, or the antegrade wire can enter the retrograde microcatheter by aligning the two microcatheters. Instead, in the case of true ostial, the wire could be advanced out of the ostium in the aorta and caught by a snare [55].

## 5. CTO Complications

CTO PCIs present higher complication rates than standard PCIs [4]. CTO-PCI complications can be categorized based on the onset time into acute and long-term, or according to the site, as cardiac and extracardiac complications [56]. They include death, emergency cardiac surgery, acute myocardial infarction, cardiac tamponade, coronary and aortic dissection, coronary perforation, thromboembolism, side branch occlusion, equipment entrapment/loss, vascular complications, major bleeding, stroke, contrast-induced acute kidney injury and radiation skin injury [57]. Identifying patients who are more likely to have procedural complications is essential to stratify the cumulative risk. Several clinical, angiographic, and technical characteristics are related to a greater risk of periprocedural complication of CTO-PCI: elderly, chronic kidney disease (CKD), retrograde and anterograde dissection/re-entry approach, and use of rotational atherectomy, which are associated with a higher risk of coronary perforations [58]. In this context, the PROGRESS-CTO complications score has been developed to predict the risk related to a procedure of CTO recanalization. It includes three parameters: age ≥ 65 years, lesion length > 23 mm, and use of a retrograde approach [59].

### 5.1. Acute Myocardial Infarction and Periprocedural Myocardial Injury (PMI)

Acute myocardial infarction during the attempt of CTO recanalization is not related to a specific technique, but it may be the consequence of several mechanisms. Dissection of a proximal vessel, deep intubation of the guiding catheter, aggressive wire utilization, or repetitive contrast media injection could limit distal flow in the vessel and cause acute ischemia. Thrombus or air embolism, which can occur during long-lasting procedures, is another possible mechanism responsible for severe ischemia and myocardial infarction [60].

Periprocedural myocardial injury is common after CTO-PCI, but the threshold of clinically relevant myocardial injury remains controversial; in addition, the use of high-sensitivity cardiac troponin significantly increases the prevalence of procedural myocardial injury, although it may be overly sensitive for discriminating prognostic impact. A recent analysis of 13 452 patients suggested that long-term mortality was not associated with any level of troponin elevation. Similarly, in a large study including 5626 patients undergoing elective CTO-PCI, post-procedure troponin T elevations were not independently predictive of adverse outcomes [61].

### 5.2. Coronary Perforation

Coronary perforation is a rare but life-threatening complication that can occur during CTO-PCI and dramatically evolve into cardiac tamponade and severe arterial hypotension, requiring immediate pericardiocentesis. The estimated incidence is about 2.6–4.8%, with a 5-fold increase in 30-day mortality [62,63]. According to Ellis’ classification (Table 4), which is the most widely used in clinical practice, there are three different types of coronary artery perforations that correspond to progressive grades of severity. We can alternatively classify coronary perforations into large (proximal), distal, and collateral vessels (septal or epicardial) perforations depending on the anatomical site. However, independent of severity and location, the first step in case of perforation is to inflate a balloon at low pressure (6–8 atm) at the site of the perforation in order to stop the extravasation [56]. In some patients, the dilatation of the balloon for a maximum of 15 min can be sufficient to close the perforation. In other cases, we have to adopt different strategies according to the location of coronary perforation. Large vessel perforations are usually treated with covered stents, possibly with the guide of intracoronary imaging [64]. Distal vessel perforation is usually related to the antegrade wiring after CTO crossing, and it can be prevented by dual arterial injection (antegrade and retrograde), showing vessel anatomy beyond the distal cap of the lesion. This type of perforation is typically treated with coil or fat embolization. Alternatively, we can use it for embolization of microspheres/beads or thrombin in case of persisting extravasation [60]. Collateral vessel perforation is a potential complication of the retrograde approach. Septal collateral perforations are generally self-limiting and do not need specific treatment, except for rare cases evolving in septal or right ventricular wall hematomas [65,66]. On the other hand, epicardial collateral perforations are more dangerous compared to septal collateral perforations, and for this reason, they should be treated with embolization from both sides to avoid prolonged pericardial [56]. Independently of their location, immediate pericardiocentesis is needed in case of cardiac tamponade, and in the rare case of persisting bleeding, cardiac surgery is required to repair the leakage [60].

### 5.3. Donor Vessel Injury

Donor vessel injury is another serious complication occurring during CTO-PCI because it is associated with severe ischemia and hemodynamic decompensation, requiring high doses of vasopressors and/or mechanical hemodynamic support. It can be induced by catheter management, especially in long retrograde procedures [67], or it can be associated with coronary spasm [58]. Donor vessel thrombosis is principally associated with long CTO PCIs, blood stasis, or suboptimal activated clotting time (ACT) [67]. To reduce the risk of this catastrophic complication, it is always recommended to advance a protection guidewire in the donor’s vessel at the beginning of the procedure; if it has a significant stenosis, it should be treated before starting CTO-PCI. In addition, maintaining the ACT above 300 s for antegrade CTO-PCI and above 350 s for retrograde CTO-PCI, with a check every 30 min, is strongly recommended to reduce the risk of catheter and vessel thrombosis [67]. Coronary spasm, a potential consequence of deep catheter engagement, often requires intracoronary infusion of vasodilators (es. Nitroglicerine, Adenosine, Nitroprusside) if it does not resolve spontaneously. If dissection occurs, balloon inflating with or without stent implantation is often needed, while vessel thrombosis can be treated with thrombectomy and/or cangrelor and glycoprotein IIb/IIIa inhibitor infusion [67].

### 5.4. Side Branch Occlusion

Side branch occlusion is another possible complication, especially during subintimal dissection/re-entry approach, and it has been associated with a high risk of post-PCI myocardial infarction [68]. It can be prevented by side branch wiring before starting CTO-PCI. Additionally, intracoronary imaging can be useful in detecting the mechanism of side-branch occlusion and assisting with side-branch recanalization [56].

### 5.5. Device Loss

Stent loss is an infrequent complication of CTO PCIs, occurring in 0.32% of cases, especially in severe tortuous or calcific lesions. It can be avoided by adequate lesion preparation before stent implantation. However, retrieval of the lost stent is not always necessary. In some cases, stent crushing results in less danger and more accessibility compared to retrieval if the lost stent is not located in the left main coronary artery or a major bifurcation. If retrieval is needed, there are several methods: the small balloon technique (advancing a balloon through the stent, inflating the balloon, and withdrawing the stent), loop snare, biliary forceps, and basket retrieval device [56].

### 5.6. Iatrogenic Aortic Dissection

Dissection of the ascending aorta could result from different mechanisms, such as damage caused by the management of a guide catheter, powerful contrast injection with a ‘wedged’ guiding catheter, or retrograde dissection propagation from the proximal or ostial segment of the coronary artery. Aortic dissection during attempted CTO-PCI of the right coronary artery is more frequent than the left coronary procedure [67]. Dissection may be limited to the ipsilateral coronary sinus of Valsalva (type I), may extend to the proximal ascending aorta (type II), or could dramatically expand over the ascending aorta (type III). A significant procedural risk factor is the deep intubation of guiding catheters, especially in the case of aggressive catheters. On the contrary, using catheters with side holes can reduce the incidence of aortic dissection.

When the dissection is limited to the coronary sinus or proximal ascending aorta, conservative treatment is suggested, with strict monitoring of vital signs and dissection progression by computed tomography or echocardiography. In some cases, implantation of a stent at the coronary ostium is needed to confine the dissection. Emergent surgery is mandatory in case of progression to ascending aorta or in case of new evidence of aortic regurgitation or pericardial effusion [60].

### 5.7. Vascular Access Complication

CTO—PCI often requires dual arterial access, with the use of larger sheath diameters than those usually used during common PCI. These conditions increase vascular complications rate by 0.5–1.5% [62]. As known, radial access is associated with lower adverse cardiac events and lower access-related bleeding than femoral access and, for this reason, is the standard vascular access during non-CTO PCI [69]. The development of thin-walled sheaths made complex PCIs feasible from the radial access with low complication rates. On the other hand, 8 French catheters may be needed due to technical aspects, and in these cases, femoral access is the most practical way [62].

### 5.8. Contrast-Induced Nephropathy

CTO-PCIs are associated with a higher risk of contrast-induced nephropathy (CIN) than common PCIs, mainly because of the high contrast volume needed [70]. There are many available modalities to prevent nephropathy, such as the use of N-acetyl-cysteine, discontinuation of nephrotoxic drugs (i.e., ACE-inhibitors, antibiotics, cyclosporine, metformin) a few hours before the procedure (according to plasma half-time). Nevertheless, the most important prevention strategies are a strict limitation of contrast amount and adequate hydration with saline solution administration (1 mL/kg for 6 h before and 12 h after the procedure), even in patients without CKD [66]. Ideally, the ratio of contrast medium to creatinine clearance should be less than two or three in patients with CKD [42,71].

### 5.9. Radiation Skin Injury

Radiation skin injury could be a deterministic and dose-dependent complication of the CTO procedure, and it includes different clinical presentations such as temporary erythema, permanent epilation, and late dermal necrosis [60]. Skin injury may occur with a skin entry dose as low as 2 Gy and invariably after 5 Gy [72]. Several methods allow for reducing the maximum cumulative skin dose during CTO procedures: use of pulsed fluoroscopy, utilization of extra-shielded x-ray tubes, and latest-generation equipment with extra beam filtering, and choice of low-dose settings [60]. In addition, it is suggested that maximum cumulative absorbed doses that exceed 1 Gy should be recorded in the patient record, and there should be a patient follow-up procedure for such cases [73].

## 6. Conclusions

Although no RCT has yet demonstrated an improvement in mortality in patients undergoing CTO-PCI, this remains a suitable therapeutic option in patients who remain symptomatic despite OMT or in those with evidence of myocardial ischemia in the occluded coronary artery distribution area during a stress test. Moreover, with the improvement of revascularization techniques and prevention strategies, when CTO-PCI is performed by experienced operators, the rate of complications has been significantly reduced. In conclusion, a better patient selection and anatomical characterization, evolving expertise, technical progress, and tailored approach will further improve the clinical benefit of CTO recanalization and the future widespread of these procedures.

## Figures and Tables

**Table 1:** Randomized clinical trials studying chronic total occlusion recanalization vs. optimal medical therapy.

Trial	Intervention	Population and Principal Baseline Characteristics	Endpoints	Results
**REVASC**	CTO PCI vs. OMT	205 stable patients OMT group (N 104): median age 68 years, N 90 males, N 31 diabetes, N 93 hypertension, N 21 current smokers, N 38 previous MI, N 61 3-vessel disease, median SYNTAX score 16 CTO PCI group (N 101): median age 65 years, N 91 males, N 32 diabetes, N 81 hypertension, N 23 current smokers, N 39 previous MI, N 53 3-vessel disease, median SYNTAX score 14	Primary: change in SWT in the CTO territory assessed by CMR Secondary: improvement of regional wall motion and changes in left ventricular volumes and ejection fraction; MACE at 12 months	No benefit of CTO PCI in terms of the primary endpoint, SWT, or other indexes of LVF. Significant reduction of MACE at 12 months in CTO PCI group (driven by repeat revascularization)
**EXPLORE**	CTO PCI vs. OMT	304 STEMI patients with CTO in non IRA artery OMT group (N 154): median age 60 years, N 126 males, N 25 diabetes, N 69 hypertension, N 76 current smokers, N 24 previous MI, N 67 3-vessel disease, MI median SYNTAX score 29 CTO PCI group (N 148): median age 60 years, N 131 males, N 22 diabetes, N 59 hypertension, N 77 current smokers, N 19 previous MI, N 62 3-vessel disease, median SYNTAX score 29	Primary: LVEF and LVEDV on CMR after 4 months	No differences in terms of LVEF, LVEDV, and 4-month MACE. LVEF significantly higher in LAD CTO subgroup treated with PCI
**EURO-CTO**	CTO PCI vs. OMT	396 symptomatic patients OMT group (N 137): median age 64.7 years, N 118 males, N 139 diabetes, N 98 hypertension, N 92 smokers, N 25 previous MI, N 24 3-vessel disease CTO PCI group (N 259): median age 65.2 years, N 215 males, N 85 diabetes, N 189 hypertension, N 190 current, N 59 previous MI, N 66 3-vessel disease	Primary: change in QoL at 12 months assessed by SAQ Secondary: MACE, stent thrombosis, cerebrovascular events, hospitalisation for cardiac reasons	Significant improvement of symptoms in CTO PCI group compared to OMT alone. No difference in MACE
**IMPACTOR-CTO**	CTO PCI vs. OMT	72 patients with isolated RCA CTO and stable angina	Primary: evaluation of ΔMIB (decrease in MIB from baseline to 12 month control) Secondary: changes in 6 min walking test, QoL, and MACE at 12 months.	ΔMIB was significantly higher in the PCI group compared to the OMT group. 6-min walk distance and QoL improved in PCI group. No significant difference in MACE between the two groups
**DECISION-CTO**	CTO PCI vs. OMT	815 patients with stable angina or ACS (not STEMI) OMT group (N 398): median age 62.9 years, N 319 males, N 134 diabetes, N 238 hypertension, N 102 current smokers, N 34 previous MI, N 128 3-vessel disease, median SYNTAX score 20.8 CTO PCI group (N 417): median age 62.2 years, N 344 males, N 132 diabetes, N 262 hypertension, N 125 Current smokers, N 45 previous MI, N 127 3-vessel disease, median SYNTAX score 20.8	Primary: composite of death from any cause, MI, stroke or any revascularisation) Secondary: individual components of the primary endpoint, bleeding, stent thrombosis and QoL (EQ-5D and SAQ)	No differences in terms of primary and secondary outcomes in the two groups
**COMET-CTO**	CTO PCI vs. OMT	100 patients with angina and/or evidence of myocardial ischaemia OMT group (N 50): median age 63 years, N 44 males, N 18 diabetes, N 43 hypertension, N 14 current smokers, N 35 previous MI, median SYNTAX score 9.87 CTO PCI group (N 50): median age 61 years, N 38 males, N 14 diabetes, N 43 hypertension, N 16 current smokers, N 29 previous MI, median SYNTAX score 10.79	Primary: evaluation of QoL by SAQ Secondary: all-cause mortality and MACE (non-fatal MI, recurrent revascularization with PCI or CABG).	Significant improvement in SAQ in CTO PCI group, while no significant differences in SAQ scores in the OMT group

ACS: acute coronary syndrome; CABG: coronary artery bypass graft; CMR: cardiac magnetic resonance; COMET-CTO: Randomised Controlled Comparison of Optimal Medical Therapy with Percutaneous Recanalization of Chronic Total Occlusion; CTO: chronic total occlusion; DECISION-CTO: Drug-Eluting Stent Implantation Versus Optimal Medical Treatment in Patients With Chronic Total Occlusion; EQ-5D: EuroQol 5 dimensions questionnaire; EXPLORE: Evaluating XIENCE and Left Ventricular Function in Percutaneous Coronary Intervention on Occlusions After ST-Segment Elevation Myocardial Infarction; IMPACTOR-CTO: Impact on Inducible Myocardial Ischemia of PercutAneous Coronary InTervention versus Optimal Medical TheRapy in Patients with Right Coronary Artery Chronic Total Occlusion; IRA: infarcted related artery; LAD: left anterior descending; LVEDV: left ventricular end-diastolic volume; LVEF: left ventricular ejection fraction; LVF: left ventriculr funztion; MACE: major adverse cardiac events; MI: myocardial infarction; MIB: myocardial ischemia burden; OMT: optimal medical therapy; PCI: percutaneous coronary intervention; QoL: quality of life; REVASC: A Randomized Trial to Assess Regional Left Ventricular Function After Stent Implantation in Chronic Total Occlusion; RCA: right coronary artery; SAQ: Seattle Angina Questionnaire; STEMI: ST-segment elevation myocardial infarction; SWT: segmental wall thickening.

**Table 2:** Chronic total occlusion complexity scores.

Score	Parameters
**J-CTO**	Blunt stump, calcification, 1 bend >45° within the occlusion, length >20 mm, prior failed CTO PCI attempt
**PROGRESS-CTO**	Proximal cup ambiguity, moderate or severe proximal tortuosity (2 bends >70° or 1 bend >90° proximal to the occlusion), circumflex CTO, absence of interventional collaterals
**CASTLE**	Previous CABG, age ≥70 years, blunt stump, severe tortuosity (≥2 pre-occlusive bends >90° or ≥1 bend >120°), length of the occlusion ≥20 mm, severe calcifications
**RECHARGE**	Previous CABG, blunt stump, calcifications, tortuosity (1 bend ≥45° within the occlusion), length of the occlusion ≥20 mm, diseased distal landing zone
**CL**	Previous CABG, previous MI, severe calcifications, length of the occlusion ≥20 mm, non-LAD location, blunt stump
**ORA**	Age ≥75 years, ostial location, collaterals Rentrop <2
**Ellis score**	Proximal or retrograde >90° bend, proximal cup ambiguity, moderate-severe calcifications, length of occlusion > 10 mm, poor target vessel, ostial CTO location

CASTLE: coronary artery bypass graft, age, stump, tortuosity, length, the extent of calcification; CL: clinical and lesion-related score; CTO: chronic total occlusion; J-CTO: Japanese Multicenter CTO Registry score; LAD: left anterior descending artery; MI: myocardial infarction; ORA: ostial location, Rentrop grade, age score; PCI: percutaneous coronary intervention; PROGRESS-CTO: Prospective Global Registry for the Study of Chronic Total Occlusion Intervention; RECHARGE: Registry of CrossBoss and Hybrid Procedures in France, the Netherlands, Belgium and United Kingdom.

**Table 3:** Guidewires categories for antegrade wire escalation.

Wire Categories	Plaque Type	Wire Features
**Tapered polymer-jacketed wires**	Soft tissue plaque	Extremely low coefficient of friction
**Intermediate tip-load**	Fibrous and fibrous-calcific plaques	Enhanced torqueability
**High tip-load guidewires**	Severe calcific lesions	Increased penetration force

**Table 4:** Ellis classification of coronary perforation.

Type	Features
**1**	Extraluminal crater without extravasation, or evidence of dissection
**2**	Pericardial or myocardial brush without contrast extravasation
**3**	Extravasation through a ≥1 mm perforation
**3 cavity spilling**	Extravasation through a ≥1 mm perforation into a circulatory chamber

## Data Availability

Not applicable.
